# Treatment interruption and associated factors among patients registered on drug-resistant tuberculosis treatment in Amhara regional state, Ethiopia: 2010–2017

**DOI:** 10.1371/journal.pone.0240564

**Published:** 2020-10-14

**Authors:** Mehari Woldemariam Merid, Atalay Goshu Muluneh, Melaku Kindie Yenit, Getahun Molla Kassa

**Affiliations:** Department of Epidemiology and Biostatistics, Institute of Public Health, College of Medicine and Health Sciences, University of Gondar, Gondar, Ethiopia; The University of Georgia, UNITED STATES

## Abstract

**Background:**

Drug-Resistant Tuberculosis (DR-TB) is a rising threat of the TB control program caused mainly by treatment interruption in Ethiopia. The success of the current treatment regimen for DR-TB is poor partly due to a high treatment interruption rate. Thus, this study assessed treatment interruption and associated factors among DR-TB patients.

**Methods:**

An institution-based cross-sectional study was conducted among 550 DR-TB patients who have initiated treatment from September 2010 to December 2017. Data were entered using Epi Data version 4.200 and exported to STATA version 14 for analysis. A bi-variable logistic regression model was first fitted, and variables having a p-value < 0.2 in the bi-variable analysis were entered into the multivariable logistic regression model. Crude and Adjusted Odds Ratios (COR and AOR) with 95% confidence interval (CI) were used to determine the strength of association between the treatment interruption and independent variables. Variables with p-value <0.05 in the multi-variable model were considered as statistically significant predictors of treatment interruption.

**Results:**

In this study, the prevalence of treatment interruption among patients registered on DR-TB treatment was **14.55% (95% CI: 11.83, 17.76).** Of the interrupters, the treatment interruption during the intensive and continuation phase of treatment was reported as 45% and 71.25%, respectively. Similarly, about 15% of patients had treatment interruption both during the intensive and continuation phase of treatment. The average duration of treatment interruption was 12 (±2.03 SD) and 6 (±1.2 SD) days during the intensive and continuation phase of treatment, respectively. Patients who had no treatment supporter [AOR = 1.45; 95% CI: 1.23–3.66] and developed adverse drug events [AOR = 1.60; 95% CI: 1.22–2.85] were statistically significant predictors of treatment interruption.

**Conclusions:**

Treatment interruption was low in the study setting. The presence of treatment supporter and absence of drug side effects was significantly associated with decreased occurrence of treatment interruption. Thus, patient linkage to treatment supporter and excellent pharmacovigilance are highly recommended in the study setting.

## Background

**Tuberculosis** (TB) is an infectious disease caused by *Mycobacterium tuberculosis (MTB*) bacteria which mainly affects the lungs. It ranks as the leading cause of death among infectious diseases in human history, claiming over a billion lives in the past two centuries alone [1]. The Drug-Resistant Tuberculosis (DR-TB), is an emerging public health threat mainly for developing countries including Ethiopia. Globally, 3.4% of new and 18% of previously treated TB cases had Multidrug-Resistant or Rifampicin-Resistant TB (MDR/RR-TB) [2].

In Ethiopia, over 80% of patients with drug-susceptible smear-positive TB complete their therapy or were successfully treated [2]. However, rates of successful cure or treatment completion among patients with DR-TB are lower, estimated to be 59.2% in 2018 [3]. The treatment for DR-TB is complex; including the use of highly toxic anti-tuberculosis drugs with potential adverse effects [4], longer treatment time [4, 5], elevated drug cost, and increased treatment failure [6–8]. As a result, poor compliance with anti- TB treatment especially, treatment interruption has become a major challenge for the successful treatment of DR-TB patients. The increased treatment interruption will ultimately have an undue influence on the TB control program. Hence, patients who interrupt treatment have an increased risk for mortality, acquisition of additional drug-resistance, and promote continued transmission of drug-resistant MTB strains in the community [9].

Many previous works of scholars have noted some of the characteristics associated with an increased probability of interrupting treatment among DR-TB patients [10–13]. These include basic patient-related factors such as educational status, sex, alcoholism, and cigarette smoking. Moreover, the patients’ clinical characteristics such as HIV co-infection, the presence of baseline comorbid conditions, previous TB treatment history, and drug side effects were also associated with treatment interruption [14–16].

The success of the current treatment regimen for drug-resistant tuberculosis is poor partly owing to a high treatment interruption rate. However, there is no study on treatment interruption and associated factors among DR-TB patients in the study setting. We, therefore, aimed to assess treatment interruption and associated factors among DR-TB patients in Amhara regional state. Determining the magnitude and associated factors of treatment interruption is essential to take actions targeting the risk factors identified for improving treatment adherence, thereby treatment success among patients on DR-TB. Furthermore, it will be beneficial for minimizing the expansion of transmission of drug-resistant MTB strains in the community.

## Methods

### Study design and setting

An institution-based cross-sectional study was conducted at the drug-resistant TB treatment initiation centers (TICs) in the Amhara Regional State from September 2010 to December 2017.

These TICs include; the University of Gondar Comprehensive Specialized Hospital, Boru-Meda generalized hospital, Debre-Markos referral hospital, and Woldia general hospital. A total of 640 DR-TB patients have initiated treatment in the Amhara region during the study period. From these, over 90% of DR-TB patients were started and had followed their Second Line anti-TB Drugs (SLD) in these four hospitals. The University of Gondar Comprehensive Specialized Hospital is the second-largest hospital giving clinical care and management for DR-TB in the country. It is found in the Central Gondar zone of Amhara. The other site was Boru-Meda generalized hospital which is located in the South Wello zone of the Amhara region primarily was known to give special care for Tuberculosis and Leprosy patients. Currently, it has advanced its care for DR-TB patients. Debre-Markos referral hospital is serving DR-TB patients in the Easter Gojjam Zone of Amhara that serves patients coming from the catchment. The fourth hospital is Woldia general hospital which is found in the North Wello Zone of Amhara and serves patients from the Northeaster parts of Ethiopia. All the four hospitals give services not only for patients in the catchment area but also to those who come from the neighboring Regional states (Tigray, Afar, and Benshagul Gumuz) of Ethiopia. The drugs used in this study were Levofloxacin (Lfx), Capreomycin (Cm), Ethionamide (Eto), Prothionamide (Pto), Cycloserine (Cs), Ethambutol (E), Pyrazinamide (Z) [17]. Accordingly, patients were taking one of the following regimens; (1) E-Z-Cm-Lfx-Eto-Cs, (2) Z-Cm-Lfx-Eto-Cs, (3) Z-Cm-Lfx-Pto-Cs. These regimens were given for a similar duration of 18 to 24 months of treatment.

### Population and sample

The source population was all DR-TB patients in the Amhara regional state while the study population ware DR-TB patients registered and followed their treatment in the four (University of Gondar, Boru-Meda, Debre-Markos, and Woldia) DR-TB treatment centers of Amhara Regional State, Ethiopia. All bacteriologically confirmed adult DR-TB patients who initiated treatment during the study period were considered. In the four selected hospitals, there were a total of 582 patients who initiated DR-TB treatment from September 2010 to December 2017 in the study setting. Of these, we included 550 DR-TB patients in the analysis who had complete data on treatment interruption and some of the key independent variables.

### Data collection and variables of the study

Data were extracted from patient charts, registration books, and computer databases using standardized data abstraction checklist. The records to be reviewed were identified using patient medical registration numbers. The data were collected by eight BSc degree graduate nurses and four health officers under the close supervision of the principal investigator. Trained data collectors reviewed and extracted data from patient medical charts and computer databases. Data were checked for any inconsistencies, coding errors, out of range values, completeness, accuracy, clarity, missing values, and appropriate corrections were made by the principal investigator consistently daily.

**A treatment supporter** is one who is involved in providing treatment support for the patients which has the key to success for DOTs and the whole duration of anti-TB treatment. The available treatment supporter options include health facility-based workers (HFW) i.e. health staff members working at the treatment centers; community health workers (CHW) i.e. any person formally associated with the health services and living close to the patient’s residence; community volunteers (CVT) like a decent person selected from the community e.g. teachers, religious leaders, neighbors, co-workers, and friends, etc [18]. A family member or any person who is willing to help and is accepted by the patient and answerable to the health services can also be a treatment supporter [19].

#### Treatment interruptions/missed doses

Treatment interruption was defined as any time that a patient missed a prescribed dose of DR-TB treatment for at least 1 day but returned to treatment for less than two consecutive months [20]. In this study, it was dichotomized as 1 = “yes” and 0 = “no” as an outcome variable.

As an independent variable, different characteristics at baseline were assessed from the medical registration documents of the patients. The first characteristic assessed was socio-demographic which included age, sex, occupational status, educational status, marital status, residence, and housing conditions. Concerning housing conditions, homeless were defined as patients who lived in streets or lacked fixed, regular, and adequate night-time residence. The second characteristics were behavioral components. These include smoking and alcohol drinking status. **Cigarette smoking**: was recorded by asking respondents whether they have ever smoked a cigarette in life history. It was dichotomized by 1 (Yes i.e., smoke cigarettes) and 0 (No smoke cigarettes). **Alcohol consumption**: was recorded by asking respondents whether they have ever drunk alcohol or not. It was dichotomized by 1 (Yes i.e., drink alcohol) and 0 (No drinking alcohol). We also collected treatment-related and clinical characteristics, which included Human Immunodeficiency Virus (HIV) co-infection, presence of baseline comorbid conditions, TB treatment history, history of injectable anti-TB drugs, and experience of drug adverse event. **An adverse event** (AE) is defined as any untoward medical occurrence that may present in a TB patient during treatment with a pharmaceutical product, but which does not necessarily have a causal relationship with the treatment [21].

Bacteriologically confirmed DR-TB was defined as patients who have a positive MTB result either by smear staining or Xpert MTB/RIF or Line probe assay or culture, and resistant to at least one anti-TB drug. Multidrug-resistance (MDR-TB): TB resistant to at least for both Isoniazid and Rifampicin. Rifampicin Resistant TB (RR-TB): TB resistant to Rifampicin detected using phenotypic or genotypic methods, with or without resistance to other anti-TB drugs except for Isoniazid [22].

## Data processing and analysis

Data were checked for completeness and entered using Epi-Data version 4.2.00 and exported to STATA version 14 for cleaning, coding, recoding, and analysis. Categorical variables were summarized by counts and percentages, and the differences among categories were compared using Pearson chi-square (X^2^). Continuous variables were described either using mean with Standard Deviation (SD) if it is normally distributed or median with Inter-Quartile Range (IQR) if the distribution becomes skewed. The binary logistic regression model was fitted by considering treatment interruption as an outcome variable. Model adequacy was assessed by the Hosmer-Lemeshow goodness of fit test. A bi-variable logistic regression model was first fitted, and variables having a p value of < 0.2 in the bi-variable analysis were entered into the multivariable logistic regression model. Crude and Adjusted Odds Ratios (COR and AOR) with 95% Confidence Interval (CI) were used to determine the strength of association between the treatment interruption and independent variables. Variables with p-value < 0.05 in the multi-variable model were considered as statistically significant predictors of treatment interruption.

## Ethics approval and consent to participate

Ethical clearance was obtained from the Institutional Review Committee of the University of Gondar College of medicine and health science. Permission letter was also obtained from the University of Gondar Comprehensive Specialized Hospital’s administration, and oral permission was also obtained from respective TICs TB ward focal person to use the data for this study. The name or any other identifier was not recorded on the questionnaire and all information is taken from the chart was kept securely in locked cabinets.

## Results

### Socio-demographic, behavioral, clinical, and treatment-related characteristics

A total of 582 confirmed DR-TB patients were registered and started on DR-TB treatment during the study period. Among these, 550 (94.5%) patients had complete records and included in the analysis. The mean age of patients was 31.4 (SD±12.36) years. More than half (52.73%) of patients were dwellers of an urban area, and 56.73% were male; about half (50.64%) were married. Nearly one-third (29.98%) of the patients were not attended school. Almost two-thirds (65.08%) of the patients were known to have some kind of adverse drug event and about 28.36% of the patients were HIV co-infected. The majority of the patients 465 (84.55%) had treatment supporter (**Table 1).**

**Table 1:** Socio-demographic, treatment-related, behavioral, and clinical characteristics of DR- TB patients in Amhara regional state, Ethiopia: 2010–2017 (N = 550).

**Variables**	**Categories**	**Frequency**	**Percept**
**Sex**	Male	312	56.73
	Female	238	43.27
**Residence**	Urban	290	52.73
	Rural	260	47.27
**Educational status**	attended school	383	70.02
	Not attended school	164	29.98
**Occupation**	Working	393	71.98
	Not working	153	28.02
**Marital status**	Married	278	50.64
	Not married	271	49.36
**Housing condition**	Have home	424	92.37
	Homeless	35	7.63
**Have a treatment supporter**	No	85	15.45
	Yes	465	84.55
**History of TB treatment for first-line drugs**	No	84	15.27
	Yes	466	84.73
**History of cigarette smoking**	No	474	86.18
	Yes	76	13.82
**History of alcohol drinking**	No	460	84.25
	Yes	86	15.75
**HIV co-infection**	No	394	71.64
	Yes	156	28.36
**Comorbid conditions**	Absent	465	84.55
	Present	85	15.45
**Experienced adverse drug event**	No	356	65.08
	Yes	191	34.92

## Treatment interruption

In this study, the prevalence of treatment interruption among patients registered on DR-TB treatment was **14.55% (95% CI: 11.83, 17.76).** Among the treatment interrupters, 45% and 71.25% were interrupted their treatment during the intensive and continuation phase of treatment, respectively. Moreover, about 15% of the patients had treatment interruption both during the intensive and continuation phase of treatment. The average duration of treatment interruption was 12 (±2.03 SD) and 6 (±1.2 SD) days during the intensive and continuation phase of treatment, respectively.

## Factors associated with treatment interruption

Findings from the bi-variable binary logistic regression analysis noted that female sex and not married were protective of treatment interruption with odds ratio less than 1 whereas not attended school, not working, had no treatment supporter, had previous TB treatment, had adverse drug event, and had comorbidities were risk factors for treatment interruption with an odds ratio greater than 1. However, in the multivariable binary logistic regression analysis, only patients who had treatment supporter and experienced adverse drug events remained significantly associated with increased occurrence of treatment interruption **(Table 2)**. After controlling the confounding effect of sex, educational status, occupation, marital status, previous treatment history, and comorbid condition, patients who had no treatment supporter were 1.45 times more likely to interrupt treatment compared to those who had treatment supporter (AOR = 1.45; 95% CI: 1.23–3.66). Similarly, patients who experienced any drug side effects were 1.60 times more likely to interrupt treatment compared to their counterparts (AOR = 1.60; 95% CI: 1.22–2.85).

**Table 2:** Bi-variable and multi-variable binary logistic regression analysis of factors associated with treatment interruption among DR-TB patients in Amhara regional state, Ethiopia: 2010–2017 (N = 550).

**Variables**	**Treatment interruption**		**COR (95% CI)**	**AOR (95% CI)**
	Yes (%)	No (%)		
Age in years			0.98 (0.96, 1.01)	1.01 (0.97,1.02)
Sex				
**Male**	55 (68.75)	257 (54.68)	1	1
**Female**	25 (31.25)	213 (45.32)	0.55 (0.33, 0.91)	0.66 (0.38,1.15)
Educational status				
**Attended school**	64 (80)	148 (31.69)	**1**	**1**
**Not attended school**	16 (20)	319 (68.31)	1.54 (0.30, 0.96)	0.79 (0.42,1.15)
Occupation				
**Working**	45 (58.44)	348 (74.2)	**1**	**1**
**Not working**	32 (41.56)	121 (25.8)	2.05 (1.24, 2.37)	1.47 (0.85, 2.35)
Marital status				
**Married**	26 (32.5)	252 (53.73)	1	1
**Not married**	54 (67.5)	217 (46.27)	0.43 (0.26,0.70)	1.60(0.89,2.86)
Treatment supporter				
**Yes**	63 (78.75)	402 (85.53)	1	1
**No**	17 (21.25)	68 (14.47)	**1.60 (0.88, 2.89)**	**1.45(1.23, 3.66)**
Have previous TB treatment				
**Yes**	64 (80)	402 (85.53)	1	1
**No**	16 (20)	68 (14.47)	1.57 (0.89,2.76)	1.58(0.87,2.88)
Had drug adverse event				
Yes	59 (73.75)	297 (63.6)	**1.16 (0.94, 2.74)**	**1.6 (1.22,2.85)**
**No**	21 (26.25)	170 (36.4)	1	1
Comorbid conditions				
**Present**	13 (16.25)	72 (15.32)	1.01 (0.53, 1.90)	0.9 (0.46, 1.77)
**Absent**	67 (83.75)	398 (84.68)	1	1

## Discussion

In this study, the overall proportion of treatment interruption among patients registered on DR-TB treatment was **14.55% (95% CI: 11.83, 17.76).** Out of 80 patients interrupted treatment, 36 (45%) were found to occur during the intensive phase and about 57 (71.25%) during the continuation phase of treatment. This finding was in agreement with some studies conducted in Ethiopia, Ghana, and India [16, 23, 24]. This could be explained in that patients might feel cured due to the disappearance of symptoms in the continuation phase of treatment and tended to interrupted treatment [24].

The magnitude of treatment interruption in our study was found to be lower compared to different studies conducted so far. For instance, the present finding was lower compared to the studies done in India [25], Nigeria [26], the Philippines [9], China [27], and South Ethiopia [28]. There may be several reasons for the observed difference in the prevalence of treatment interruption between ours and others. For instance, the lower proportion of treatment interruption in our study could be due to the use of standardized drug regimen, aggressive treatment of adverse drug events, and provision of free treatment in the study setting [29]. Besides, the collaborative work of the Federal Ministry of Health, Ethiopia (FMOH) with different partners such as the Global Health Committee (GHC) project, Global fund, and challenge TB have played an essential role through the provision of food baskets, financial aid, and social support thereby reducing treatment in the region [30]. This was supported by our finding where the presence of social supporter for the patient and the absence of drug side effects were associated with decreased occurrence of treatment interruption.

However, the magnitude of treatment interruption in our study was higher than studies from China [12], and Ethiopia [13]. The definition used across studies was one of the possible reasons for the difference in treatment interruption. For example, the national retrospective study done in Ethiopia included all patients who missed any single dose during the treatment course.

## Factors associated with treatment interruption

In the present study, the drug side effect was one of the independent predictors for treatment interruption among DR-TB patients. Hence, patients who experienced any adverse drug event were one and a half times likely to have treatment interruption compared to their counterparts. This was supported by studies done elsewhere [15, 23, 31, 32]. Pieces of evidence have indicated that the drug side effect was the most common reason for stopping treatment [15, 33]. Similarly, a study from South India has found drug-related problems like nausea, vomiting, and dizziness to be the leading cause of treatment interruption in tuberculosis patients [34]. Furthermore, a prospective study in South Africa revealed that about 39.4% of the patients had discontinued treatment due to presumed linezolid associated toxicity [35].

Adverse drug reactions are expected to influence adherence to treatment particularly for DR-TB patients where they have to take more toxic and longer anti-TB drugs [36]. Subsequently, patients might fail to resist the discomfort brought by the drugs and decided to interrupt treatment. Furthermore, patients would perceive that the health benefit they could get from taking the treatment was not worth suffering the negative side effects of the medicines. Long term goals of cure and recovery from the diseases were disregarded for the immediate goal of seeking relief from the discomfort brought about by the adverse effects of the medications.

The other significant independent predictor for treatment interruption was the presence of a treatment supporter for the patient. In our study, patients who had no treatment supporter were one and a half more likely to have treatment interruption compared to those who had a treatment supporter. This was consistent with the findings from Nigeria [14] and Ghana [16] which reported lower treatment interruption and better treatment outcomes among patients supervised by treatment supporters. This can be justified by the fact that the closer the treatment supporter lives to the patient, the better the treatment adherence and successful outcome [37]. Moreover, most of the patients may have extended family with good social life, fun, good economic, and moral support before the incidence of disease and while they feel the pinch of it they may become hopeless that insist them to interrupt the treatment.

## Limitation of the study

We are confident that our study is strong but it is not without limitations. Firstly, as we have relied on secondary data, we could not access data on the number and gaps of treatment interruption, and date of each interruption which would have been very important to characterize the pattern of treatment interruptions and to link with treatment success. Besides, we were unable to address all potential variables like distance from TICs, and some key variables related to the health care facilities and health care provider worker.

## Conclusions

Treatment interruption was low in the study setting. The presence of treatment supporter and absence of adverse drug effects was significantly associated with decreased occurrence of treatment interruption. Thus, patient linkage to treatment supporter and excellent pharmacovigilance are highly recommended in the study setting.

## Supporting information

S1 Data (DTA)

## Abbreviations

TB: Tuberculosis
DR-TB: Drug-Resistant Tuberculosis WHO: World Health Organization
GHC: Global Health Committee
TIC: Treatment Initiating Centres
AIDS: Acquired Immune-Deficiency Syndrome
HIV: Human Immune Virus
